# Hypertension: Constraining the Expression of ACE-II by Adopting Optimal Macronutrients Diet Predicted via Support Vector Machine

**DOI:** 10.3390/nu14142794

**Published:** 2022-07-07

**Authors:** Mohammad Farhan Khan, Gazal Kalyan, Sohom Chakrabarty, M. Mursaleen

**Affiliations:** 1 Digby Stuart College, University of Roehampton, London SW15 5PU, UK; farhan7787@gmail.com; 2 Department of Pathology, School of Medicine and Health Sciences, University of North Dakota, Grand Forks, ND 58202, USA; gazaljii@gmail.com; 3 Department of Electrical Engineering, Indian Institute of Technology Roorkee, Roorkee 247667, India; sohom.chakrabarty@ee.iitr.ac.in; 4 Department of Medical Research, China Medical University Hospital, China Medical University (Taiwan), Taichung 40402, Taiwan

**Keywords:** SVM, COVID-19, feature filtration, hypertension, macronutrients

## Abstract

The recent elevation of cases infected from novel COVID-19 has placed the human life in trepidation mode, especially for those suffering from comorbidities. Most of the studies in the last few months have undeniably raised concerns for hypertensive patients that face greater risk of fatality from COVID-19. Furthermore, one of the recent WHO reports has estimated a total of 1.13 billion people are at a risk of hypertension of which two-thirds live in low and middle income countries. The gradual escalation of the hypertension problem andthe sudden rise of COVID-19 cases have placed an increasingly higher number of human lives at risk in low and middle income countries. To lower the risk of hypertension, most physicians recommend drugs that have angiotensin-converting enzyme (ACE) inhibitors. However, prolonged use of such drugs is not recommended due to metabolic risks and the increase in the expression of ACE-II which could facilitate COVID-19 infection. In contrast, the intake of optimal macronutrients is one of the possible alternatives to naturally control hypertension. In the present study, a nontrivial feature selection and machine learning algorithm is adopted to intelligently predict the food-derived antihypertensive peptide. The proposed idea of the paper lies in reducing the computational power while retaining the performance of the support vector machine (SVM) by estimating the dominant pattern in the features space through feature filtering. The proposed feature filtering algorithm has reported a trade-off performance by reducing the chances of Type I error, which is desirable when recommending a dietary food to patients suffering from hypertension. The maximum achievable accuracy of the best performing SVM models through feature selection are 86.17% and 85.61%, respectively.

## 1. Introduction

The novel pandemic coronavirus disease 2019 (COVID-19 or SARS-CoV-2) has undoubtedly created global anxiety, especially for people suffering from severe chronic diseases. Most of the recent studies concomitant to COVID-19 from China have suggested the fatalistic role of the novel virus for patients with comorbidities such as cerebrovascular diseases, diabetes, hypertension and others. One of the studies conducted on 191 confirmed cases in China asserted that, the most frequent comorbidities that were found in the nonsurviving patients were hypertension (48%), diabetes (31%) and coronary heart disease (24%) [1]. Another study conducted on a confirmed 44672 patients indicate that the overall fatality rate with preexisting comorbid conditions is 10.5% for cardiovascular disease, 7.3% for diabetes, 6.3% for chronic respiratory disease, 6.0% for hypertension and 5.6% for cancer [2]. It is worth noting that the investigations have revealed that the nonsurviving hypertensive patients were frequently treated with angiotensin converting enzyme (ACE) inhibitors [3].

Hypertension is the physical exertion of the blood on the walls of the blood vessels, and is currently one of the major concern which is aggravating the risk of fatality through COVID-19 by approximately 250% [4]. Other than the COVID-19 risk factor, the prolonged uncontrolled hypertension above 140 systolic and 90 diastolic (in mmHg) can lead to the severe health risks such as cardiovascular disease and stroke [5,6,7]. The crucial pathway that holds the tendency to regulate blood pressure as well as systemic vascular resistance is the renin–angiotensin–aldosterone system (RAAS) [8,9].

To counter the problem of high blood pressure in patients, most physicians recommend the use of drugs having ACE inhibitors that have a tendency to relax blood vessels and eventually reduce blood pressure. However, prolonged use of such drugs can have severe adverse side effects such as hyperkalemia, dry cough etc. [10,11,12]. Furthermore, treating the hypertension with ACE inhibitors results in upregulation of the angiotensin-converting enzyme II (ACE-II) [13], which could facilitate infection with COVID-19.

One of the alternatives to prevent the side effects of the drugs is to focus on the intake of optimal macronutrients [14,15]. Clinical trials such as DASH (dietary approaches to stop hypertension) [16] and omni-heart [17] have proven that certain macronutrients are responsible for naturally lowering the blood pressure through pertinent food intake. protein-rich diet, however, is rudimentary since only the proteins that can be broken into functional bioactive peptides are vital for exhibiting the antihypertensive property [18]. Food-derived peptides with antihypertensive activity are viewed as one of the major players to reduce most metabolic risks [19]. Certain bioactive peptides interact with the key enzyme ACE-I and act as ACE-I inhibitory peptides [20], thus being similar to the prescribed drugs. Nonetheless, the peptides are a more natural and milder alternative. However, experimental detection and identification of the food-derived antihypertensive peptides in diverse dietary food choices is a costly process [21].

Over the past few years, depending on the type of dataset, the machine learning methods are acting as vital tool for identifying various diseases such as diabetes, cancer, hypertension and many more [22,23,24,25,26,27]. Tapak et al. [22] has noted various machine-learning classifiers to classify diabetes with the help of various risk features. Out of various machine learning algorithms, the support vector machine (SVM) has outperformed other algorithms namely random forest (RF), neural network, fuzzy c-mean and linear discriminant analysis [22]. Similarly, Lee et al., have utilised various machine learning algorithms such as SVM, logistic regression, K-nearest neighbors, etc., to create a supervised model for detecting type 2 diabetes [23]; while Barakat et al. [24] have proposed the SVM models for predicting diabetes mellitus.

Siqueira et al. [25] have classified the mid-infrared spectroscopy prostate cancer with the help of various SVM models constituted of different kernels. The performance comparison among various kernels have indicated that the radial basis function (RBF) has outperformed linear, quadratic and polynomial functions. Dealing with the problem of noisy data, Ju et al. have developed a fuzzy SVM algorithm which can handle the erroneous data of phosphoglycerylation sites [26]. On the other hand, to classify and predict the pulse wave of hypertensive and healthy groups, Luo et al. [27] have utilised four machine learning algorithms namely AdaBoost, SVM, Gradient Boosting and Random Forest.

The investigations have revealed that inclusion of the trivial features while training the models not only increases the computational complexity of the algorithm but also adversely impacts the prediction accuracy of the model [28,29,30]. It is worth noting that usually the machine learning tools perform efficiently under the circumstances where the decision boundaries are well-defined.

However, the high dimensional biological systems are prone to noise or disturbances that may lead to erroneous data points [31,32]. Hence, to control the level of uncertainty while developing a predictive model, it is desirable to integrate the nontrivial feature selection methods as pre-processing tools that can filter trivial features and classify the focal point of a disease more efficiently. The core objective behind feature selection is to reduce the unnecessary features that do not hold sufficient information for classification.

It is worth noting that most of the machine learning algorithms that can identify the food-derived antihypertensive peptides are diverse in nature and are solely established through objective-based studies. The methods reported in the literature that predict antihypertensive peptides have a major limitation point out that: (a) no clear factor has been defined to differentiate and label the positive and negative classes of antihypertensive peptides, and (b) they use a trivial feature for training the computational model.

The aforementioned limitations may result in performance deterioration of computational algorithms by prioritising statistical Type I error. Moreover, while supervising a machine learning model, it is desirable to use an integrated method to predict bioactive peptides from food protein sequences which hold vital information of biological sequences in the form of physical features of the peptides.

Thus, the aim of the present study is to develop a machine-learning-based computational model that can predict the antihypertensive food peptides with better accuracy. The proposed idea is helpful in abating the frequency of drug intake and elevating the habit of a balanced diet constituting equal amount of optimal macronutrients which can control hypertension. Note that such transformation is highly desirable to constrain the upregulation of ACE-II which plays a leading role in facilitating COVID-19 infection.

This paper is organized as follows. A brief overview of materials and methods is described in Section 2. The vital role of features in enhancing the performance of the proposed SVM algorithms is explored in detail in Section 3. Section 4 interprets the findings of your study The case study of chicken egg white protein is included in Section 5, followed by concluding remarks in Section 6.

## 2. Materials and Methods

### 2.1. Antihypertensive Peptides Database

The training and testing datasets were prepared from food-derived antihypertensive peptides which are available on the weblink http://hazralab.iitr.ac.in/ahdb/index.php (accessed on 29 April 2021) [21]. The 715 total peptides used in this study have been experimentally validated to show antihypertensive activity within the course of past in vitro and in vivo studies. From the complete dataset, a total of approximately 10% of the peptides were randomly selected as a holdout sample for testing the accuracy of the machine learning model.

Note that the hypertensive sub-dataset is comprised of 136 peptides while the anti-hypertensive sub-dataset is comprised of 579 peptides of which approximately 10% of the data values from each sub-dataset (i.e., 14 hypertensive and 58 anti-hypertensive peptides) were randomly picked and reserved for testing the accuracy of the machine learning model, while the remaining 643 peptides were used to train the machine learning model. The objective behind reserving 72 peptides from the training dataset is to create a small veiled set of data points which can later be used to analyse the accuracy of the machine learning models.

The quantification of inhibition is determined by the concentration of peptide at which it inhibits 50% of the target, which is expressed as IC_50_ value. The ACE-I inhibitory activity assays have been used to obtain the experimental IC_50_ concentration of a peptide [33,34]. At lower concentrations, the peptide shows promising results of ACE-1 inhibition, while at larger concentrations, it shows negligible to no activity of inhibition. This criteria is used to distinguish anti-hypertensive peptides among the collection of peptides. Furthermore, the data related to each peptide which have been calculated as the descriptors and properties of these peptides is also available.

### 2.2. Feature Selection

A knowledge-based heuristic approach has been used to select features for the purpose of machine learning. These features are divided into two categories, namely structural features and pseudoamino acid composition (PseACC) features. The peptide sequence descriptors include amino acid composition as well as Chou’s pseudoamino acid composition for incorporation of the sequence order information [35]. With success of PseACC in the sequence-based prediction [36,37,38], it is an imperative addition to the standard composition feature vectors. The peptide structure descriptors have been formulated with molecular weight, peptide shape (R,α,β), positive charge ($q_{+}$), negative charge ($q_{-}$) and volume. These features not only encompass the three-dimensional shape and size of the peptide, they also describe the capacity of the peptide to form noncovalent interactions with the ACE-I [39]. It has been seen that if the charge of the surface of peptides is complementary to the charge on ACE-I, there will be a stronger interaction and thereby stronger inhibition capability [39].

### 2.3. Machine Learning Models

In this paper, some of the variants of well known machine learning algorithms, such as decision tree [40], logistic regression [41], SVM [42] and *k*-nearest neighbour [43] are tested for the developed antihypertensive peptides database. The leaves of the decision tree algorithm are divided into two variants which can make different level of distinctions between classes. The two variants of the decision trees which are considered in this work are fine and coarse having a maximum of 5 and 100 splits, respectively. Similarly, to train and test the SVM model, the four kernels, namely linear, quadratic, cubic and radial basis function (RBF) are chosen. On the other hand, the *k*-nearest neighbour algorithm is divided into two variants depending on the number of neighbours and distance metric. The two variants of *k*-nearest neighbour are fine (having 5 neighbours and Euclidean as distance metric) and cosine (set to 5 neighbours and cosine as distance metric). Out of all the aforementioned machine learning algorithms, the SVM model with radial basis kernel function has outperformed in terms of accuracy (refer to Table 1). Due to higher accuracy of the SVM compared to other contemporary methods, in this work, an RBF kernel SVM model is preferred for further performance optimisation.

### 2.4. Support Vector Machine (SVM) Model

The SVM model can differentiate the different classes of the peptides through optimal classification boundary by solving the dual Lagrange objective function. Consider a linear classification case, where median decision surface is separating the antihypertensive behaviour of peptides into negative and positive classes, which can be defined in the form of hyperplane $H_{0}$. Mathematically, $H_{0}$ can be defined as $\vec{w}\cdot \vec{x}+b=0$, where $\vec{w}$ is the weight vector, $\vec{x}$ is the input vector of antihypertensive data, and *b* is the bias constant. To accurately identify the classes of unknown antihypertensive data, it is necessary to maximise the width between two classes.

Now, consider two hyperplanes $H_{1}$ and $H_{2}$ for positive and negative class, respectively, such that there is no data point in between the hyperplane. Note that the supporting points of $H_{1}$ and $H_{2}$ are known as tips of SVs (support vectors). Mathematically, all *x*, $H_{1}$ and $H_{2}$ can be defined as, $\vec{w}\cdot \vec{x}+b\geq 1$ and $\vec{w}\cdot \vec{x}+b\leq -1$, respectively.

Maximising the width of the margin (*d* = $2/\left|\right|\vec{w}\left|\right|$) or distance between SVs which separates the two classes using $H_{1}$ and $H_{2}$ can be represented in terms of convex optimisation:

(1) $$\begin{array}{c} \min\;\frac{1}{2}\left|\right|\vec{w}{\left|\right|}^{2}\;s.t.\;y_{i}\left(\vec{w}\cdot {\vec{x}}_{i}+b\right)\geq 1 \end{array}$$

Note that $y_{i}$ = +1 or −1. to generalise SVM and allow errors in the training set, a slack error variable $\eta_{i}\geq 0$ is used to penalise the data points falling in the undesirable regions. The constraint defined in Equation (1) modifies to:

(2) $$\begin{array}{c} \min\;\frac{1}{2}\left|\right|\vec{w}{\left|\right|}^{2}\;s.t.\;y_{i}\left(\vec{w}\cdot {\vec{x}}_{i}+b\right)\geq 1-\eta_{i} \end{array}$$

Furthermore, to control the problem of over- and underfitting of the SVM model due to erroneous data points, a soft control variable $\hat{C}$ can be introduced in Equation (2), resulting in a quadratic optimisation problem.

(3) $$\begin{array}{c} \min\;\frac{1}{2}\left|\right|\vec{w}{\left|\right|}^{2}+\hat{C}\sum_{i=1}^{n}\eta_{i}\;s.t.\;y_{i}\left(\vec{w}\cdot {\vec{x}}_{i}+b\right)\geq 1-\eta_{i} \end{array}$$

To solve the optimisation problem, a Lagrange objective function ($L(\vec{w},\beta )$) can be introduced (where constraint β≥0) which can be defined for Equation (1) as follows:

(4) $$\begin{array}{c} L\left(\vec{w},\beta \right)=\frac{1}{2}\left|\right|\vec{w}{\left|\right|}^{2}-\sum_{i=1}^{n}\beta_{i}\left[\left(\vec{w}\cdot {\vec{x}}_{i}+b\right)y_{i}-1\right] \end{array}$$

Due to the introduction of variables $\hat{C}$ and η, $L(\vec{w},\beta )$ modifies to $L^{\prime }\left(\vec{w},\beta \right)$:

(5) $$\begin{array}{c} L^{\prime }\left(\vec{w},\beta \right)=\frac{1}{2}\left|\right|\vec{w}{\left|\right|}^{2}+\hat{C}\sum_{i=1}^{n}\eta_{i}-\sum_{i=1}^{n}\beta_{i}\left[\left(\vec{w}\cdot {\vec{x}}_{i}+b\right)y_{i}-1+\eta_{i}\right] \end{array}$$

The goal is to solve the dual Lagrange objective function $\max_{\beta \geq 0}$ $\min_{\vec{w},b}$ $L^{\prime }\left(\vec{w},\beta \right)$. To solve the dual Lagrange objective function for the optimal value of *w* and *b* (as a function of β), the partial derivatives $\partial L^{\prime }/\partial w=0$ and $\partial L^{\prime }/\partial b=0$ can be evaluated and substituted in Equation (4) which modifies the objective function to:

(6) $$\begin{array}{c} \max\;\sum_{i=1}^{n}\beta_{i}-\frac{1}{2}\sum_{i=1}^{n}\sum_{j=1}^{n}\beta_{i}\beta_{j}y_{i}y_{j}\left(\vec{x_{i}}\cdot \vec{x_{j}}\right)\\ \;s.t.\;\hat{C}\geq \beta_{i}\geq 0\;\forall i,\;\sum_{i=1}^{n}\beta_{i}y_{i}=0 \end{array}$$

to deal with the problem of nonlinear classification, the expression $\left(\vec{x_{i}}\cdot \vec{x_{j}}\right)$ modifies to $K(\vec{x_{i}},\vec{x_{j}})$, where *K* represents kernel function.

In this work, the performance of the SVM is computed for different type of kernels such as linear, quadratic, cubic and radial basis function (RBF). It has been found that, out of all, the RBF kernel gives the best performance with comparatively the highest prediction accuracy. Hence, in this work, the RBF kernel $K(\vec{x_{i}},\vec{x_{j}})$ is adopted for further analysis, which can be defined as:

(7) $$\begin{array}{c} K\left(\vec{x_{i}},\vec{x_{j}}\right)=\exp\left(-\frac{\left|\right|x_{i}-x_{j}{\left|\right|}^{2}}{2\sigma^{2}}\right) \end{array}$$

where, σ is a kernel scaling parameter. Substituting Equation (7) in Equation (6) defines the optimisation problem for the RBF kernel.

It is worth noting that the tuning variables $\hat{C}$ and σ of the RBF kernel SVM model plays a vital role in defining the final SVM model for the antihypertensive database, and it is necessary to perform a rigorous search to find the best performing pair. Investigations have revealed that in bioinformatics or computational biology analysing the importance of features prior to applying a machine learning algorithms had not been a common practise, which can dreadfully affect the performance accuracy of the machine learning models by including irrelevant features and also likely introducing a statistical Type I error.

To overcome the aforementioned limitation, the proposed algorithm is designed to extract a distinct subset of features by utilising two feature selection methods. Then, the resulting subsets are passed through fundamental operations through which both of the feature subsets can be combined and related with each other, resulting in a hybrid nontrivial feature space.

### 2.5. Nontrivial Feature Selection and Pattern of Dominance

In this section, the nontrivial features are analysed in the data space with the help of statistical analysis. The objective of this section is to analyse the extract of the pattern of dominance with the feature space which can help in reducing the statistical Type I error while predicting the antihypertensive class of unknown food-derived peptides.

We investigate the percentage of variability explained by each feature in the feature space that has been analysed with the help of singular value decomposition (SVD) [44]. The SVD is a vital tool in providing a dominant pattern within the high dimensional system, which can efficiently provide the low rank approximation of the system by decomposing the feature space (**X**) of rank *r* into three unitary matrices **U, Σ** and **V**^*T*^, which satisfies the following expression:

(8) $$\begin{array}{c} X=U\Sigma V^{T} \end{array}$$

where **X**$\in R^{m\times n}$, **U**$\in R^{m\times r}$, **Σ**$\in R^{r\times r}$, and **V**^*T*^ $\in R^{r\times n}$. Note that **Σ** is a diagonal matrix which contains nonzero eigenvalues of the feature space, i.e., **Σ**= diag$\left\{\sigma_{1},\sigma_{2},\cdots ,\sigma_{r}\right\}$, where $\sigma_{1}\geq \sigma_{2}\geq \cdots \geq \sigma_{r}$. While, the matrix **V**^*T*^ is comprised of eigenvectors whose strength of contribution in the feature space has been quantified by respective eigenvalues in matrix **Σ**. Due to the direct relation of eigenvalue matrix **Σ** with feature space matrix **X**, equating the lowest values of **Σ** to zero will result into low dimensional approximate feature space. In other words, to estimate the dominant feature(s), it is desirable to find α number of features which can efficiently preserve a higher amount of information of the actual feature space.

To estimate the nontrivial feature space subset within the complete dataset, two methods, namely MRMR [45,46] and SIDR, have been adopted. The prior algorithm discovers an optimal set of features that is mutually disparate and ranks the features according to the entropy of mutual information, while the latter method applies the Kruskal–Wallis one-way ANOVA test [47] to find the significance difference among the features. In this work, for SIDR feature filtering, the confidence levels of 1% and 5% have been considered [48].

## 3. Result

**Figure 1:**
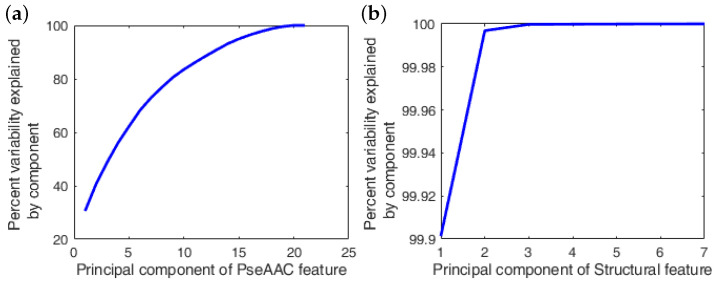
Percent variability explained or information preserved by each feature in the feature space **X**. Variability in the data by considering: (**a**) PseAAC feature; (**b**) structural feature.

In this section, the role of feature selection in estimating the relevant feature space is investigated, which helps in enhancing the accuracy of the SVM model by eliminating trivial features from the training dataset. Prior to adopting any feature selection approach, it is necessary to predict the importance or dominance of specific feature(s) in the entire feature space with the help of SVD analysis. Figure 1 demonstrates the variability explained by each feature in both the subsets of the entire feature space.

Observing Figure 1a, it can be asserted that out of the complete set of the PseAAC feature space, 13 components are able to explain 90% of the total information consisting within the entire feature space of the PseAAC. Furthermore, an additional six features are able to attain a total of ≈99.9% of the information. In contrast, considering the case of the structural feature, only one feature is able to capture >90% of the total information. Hence, it can be asserted that according to SVD analysis, the remaining features in both feature subsets are not substantially required in the classification process and can be treated as trivial features.

**Figure 2:**
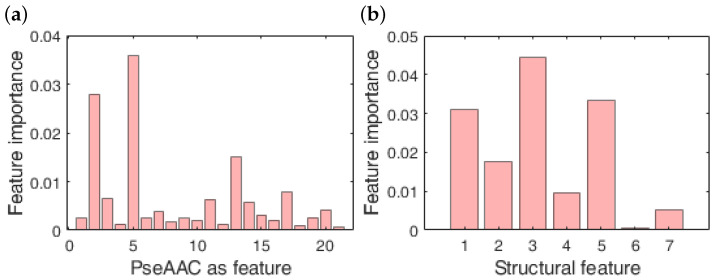
Confidence score of features represented in the form of bar graph: (**a**) feature importance of PseAAC, (**b**) feature importance of structural properties. Peaks in the graph represent higher confidence in predicting the most important feature for the classification process.

On the other hand, Figure 2 predicts the score of the features using the MRMR algorithm. The score has been estimated after dividing the overall feature space into two subsets. The first feature set consists of PseAAC as variable having a total of 21 features, while the second feature set is comprises of structural characteristics such as *R*, α, β, $q_{+}$, $q_{-}$ and volume. Figure 2a illustrates that out of all the 13 most important pseudoamino acids that have a comparatively higher MRMR score are alanine (A), cysteine (C), aspartic acid (D), phenylalanine (F), glycine (G), histidine (H), methionine (M), proline (P), glutamine (Q), arginine (R), threonine (T), tryptophan (W) and tyrosine (Y). On the other hand, α, and $q_{+}$ are the top two features in the structural subset (refer Figure 2b) that hold a relatively higher MRMR score.

In contrast, the SIDR algorithm utilises a nonparametric Kruskal–Wallis one-way ANOVA test, which has been applied on the feature space that is in the ordinal measurement scale such as the subset of positive and negative hypertensive peptides. Note that the normality of the features has been analysed using the D’Agostino–Pearson test of normality by setting a critical chi-squared value to 0.05 [47]. The *p*-value of all the features is coming out to be <0.05, resembling rejection of the null hypothesis; hence it concludes that the data is not following a normal distribution. The null hypothesis of this test is based on the assumption that the samples are drawn from same population or both samples have equal median values [49]. As per the prediction made by the SIDR algorithm in Table 2, the components within the feature space that fall in the range of confidence level of 1% are cysteine (C), glutamic acid (E), glycine (G), tryptophan (W), tyrosine (Y) and $q_{+}$. Whilst for the case of the 5% confidence level, a total of 10 features qualify for the nontriviality post.

### 3.1. Biological Significance of Nontrivial Features

In the previous section, the statistical analysis indicated the role of some vital amino acids and structural features in significantly differentiating the properties of the peptides. These features potentially assist in identifying the proteins from which antihypertensive peptides can be extracted. Food-derived peptides satisfying the characteristics of the predicted features of the peptides are immensely functional. The possible biological significance of some of the nontrivial amino acids and structural characteristics that are significantly contributing to the MRMR and SIDR algorithms in predicting the antihypertensive activities of the peptides is discussed in Supplementary File S1.

### 3.2. Performance Evaluation

**Figure 3:**
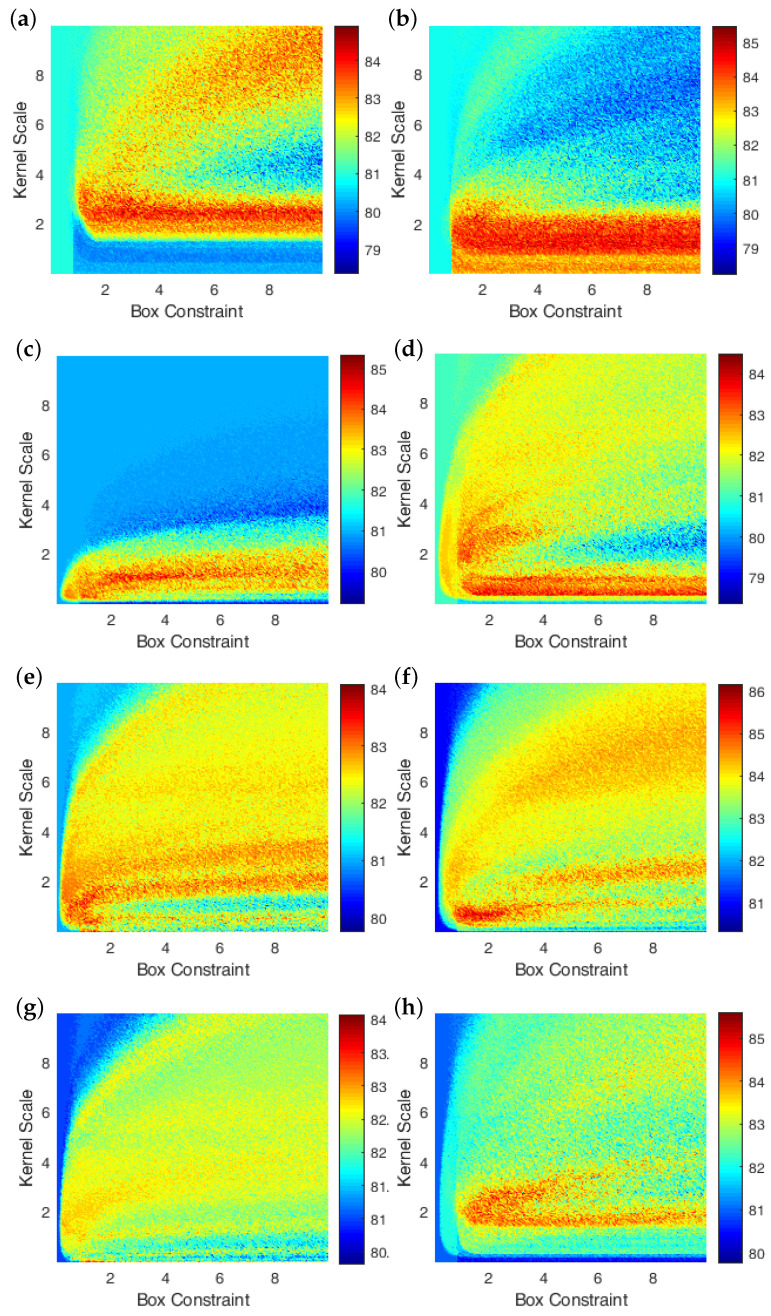
Deviation in the accuracy of the SVM model due to a variation in the feature space for systematic combinations of box constraint ($\hat{C}$) and kernel scale (σ). Using feature selection methods, the following features have been extracted for performance comparison: (**a**) all features (or reference feature space); (**b**) PseAAC features; (**c**) structural features; (**d**) features extracted from MRMR; (**e**) features extracted from SIDR (p=0.01); (**f**) features extracted from SIDR (p=0.05); (**g**) features extracted from MRMR ∩ SIDR; and (**h**) features extracted from MRMR ∪ SIDR.

To analyse the impact of dominant patterns on the estimation of antihypertensive peptides, five performance evaluation metrics have been included, namely, accuracy, area under curve (AUC), sensitivity, specificity and Matthew’s correlation coefficient (MCC). Out of all the five performance metrics, the accuracy of the SVM has been considered as the highest priority metric to estimate the best performing combination of variables $\hat{C}$ and σ. Figure 3 demonstrates the variation in the accuracy of the SVM models due to the filtration of some features from the feature space. Observing all the sub-figures of Figure 3, it can be asserted that the surface of the accuracy distribution is highly nonlinear in nature; hence it is most likely that the Bayesian optimisation routine [50] will fall into the local minina for estimating the best performing combinations of variables $\hat{C}$ and σ, which is self evident from Table 3.

Hence, instead of using an optimisation function, a systematic combination search algorithm has been performed to find the best performing combinations of $\hat{C}$ and σ. Figure 3a illustrates the reference SVM model accuracy which includes all the 28 features in the feature space and is able to achieve a maximum accuracy of 84.90% for $\hat{C}=2.8001$ and σ=2.7501. While considering core subset features, i.e., PseAAC and structural features individually can yield a maximum of 85.47% (at $\hat{C}=1.9501$ and σ=3.5001) and 84.49% (at $\hat{C}=0.5001$ and σ=0.4501), respectively, which is a bit less than reference value.

The filtering of trivial features using algorithms such asMRMR and SIDR (p=0.01) has further deteriorated the accuracy of the model and is giving comparatively less accuracy of 84.49% (for $\hat{C}=1.0001$ and σ=3.4001) and 84.07% (for $\hat{C}=1.2001$ and σ=5.4001. On the other hand, the SIDR (p=0.05) algorithm has outstandingly performed by giving the highest accuracy of 86.17% for $\hat{C}=0.7001$ and σ=1.8501. To investigate the combined effect of MRMR and SIDR algorithms, the converging and diverging features of both algorithms are opted. The intersection of MRMR and SIDR has nominated four PseAAC and one structural features, which are giving the highest accuracy of 84.07% at $\hat{C}=1.1501$ and σ=1.6501. In contrast, the union of both the aforementioned algorithms has suggested 15 PseAAC and 5 structural nontrivial features, which resulted in 85.61% of accuracy at $\hat{C}=2.6001$ and σ=2.1001.

Table 4 further elaborates the performance of various sub-feature spaces compared to the complete feature space. Note that the two best performing values are boldfaced in each metric for enhanced visualisation. From Table 4, it can be observed that SIDR (p=0.05) and MRMR ∪ SIDR include a maximum number of metrics that are giving the best performance, perhaps due to fact that both algorithms include maximum features that are biologically significant in defining antihypertensive activity of the peptide (refer to Section 3.1). The only metric that has weaker performance in the SIDR (p=0.05) algorithm is AUC, while in the MRMR ∪ SIDR algorithm it is sensitivity. It is worth noting that while predicting the antihypertensive peptide, not all metrics hold equal importance. For example, the patients suffering from hypertension are expected to take those food products that must have antihypertensive properties.

## 4. Discussion

The usefulness of applied machine learning in predicting food-derived antihypertensive peptides is critical to analyse due to existence of Type I and Type II errors during training process. Under such scenarios, the only solution is to opt a best possible trade-off which gives importance to specific error by analysing the significance of specific class. Hence, to overcome the aforementioned limitation, this study focuses on detecting positive class of food derivative peptide because it holds more importance than negative class of peptide in dealing with critical hypertensive patients. That is, the tolerance for detecting antihypertensive peptides with Type I error (false positive) is much lower than Type II error (false negative) [47], which makes sensitivity metric less impeccable than specificity metric; and with the similar logic AUC also holds higher importance during estimation of positive food derivative peptide. Observing the AUC curves of all the varying feature spaces, illustrated in Supplementary File S2, it can be asserted that the algorithm MRMR ∪ SIDR is relatively better than other feature selection algorithms and is able to report higher number of true positive cases.

The maximum attainable accuracy of the proposed SVM model in predicting the antihypertensive peptide is 86.17%, which is better than the previously existing models demonstrated in the artificial intelligence empowered web servers such as AHTpin and PAAP giving average accuracy of 78.58% and 84.73%, respectively [51,52]. Note that one of the major limitations of both the aforementioned algorithms is their comparatively lesser value of specificity metric, which is 78.89% for AHTpin and 77.65% for PAAP [52]. As stated earlier, the lesser the value of specificity is, the stronger the confidence in conducting Type I error will be. That is, it can be disastrous to recommend a dietary food to the patients suffering from hypertension in which the artificially intelligent algorithm is not reasonably confident in correctly predicting the antihypertensive property of the dietary food.

It is worth noting that when considering a trade-off between Type I and Type II errors, it is desirable to adopt an SIDR (p=0.05) algorithm for predicting the activity of the peptides. However, the chances of obtaining a reasonable trade-off value between Type I and Type II errors is lesser in our case due to lack of a big dataset. In future work, the intent is to experimentally validate large numbers of peptides so that they can be used for developing more accurate machine learning models.

## 5. Case Study of Chicken Egg White Protein

In this section, the performance of SIDR (p=0.05) and MRMR ∪ SIDR has been tested for chicken egg white protein. The ACE inhibitory peptides from chicken egg white protein have demonstrated its vital role in constraining blood pressure in vivo [53,54]. So, in this section, the food protein sequence of chicken egg white extracted from UniProt is considered for estimating the specific peptides known for its antihypertensive activity. The UniProt ID of the protein sequence is P01012 [55], which is also mentioned below.

>sp|P01012|OVAL_CHICK Ovalbumin OS=Gallus gallus OX=9031 GN=SERPINB14 PE=1 SV=2.

MGSIGAASMEFCFDVFKELKVHHANENIFYCPIAIMSALAMVYLGAKD

STRTQINKVVRFDKLPGFGDSIEAQCGTSVNVHSSLRDILNQITKPNDVY

SFSLASRLYAEERYPILPEYLQCVKELYRGGLEPINFQTAADQARELINSW

VESQTNGIIRNVLQPSSVDSQTAMVLVNAIVFKGLWEKAFKDEDTQAM

PFRVTEQESKPVQMMYQIGLFRVASMASEKMKILELPFASGTMSMLVLL

PDEVSGLEQLESIINFEKLTEWTSSNVMEERKIKVYLPRMKMEEKYNLT

SVLMAMGITDVFSSSANLSGISSAESLKISQAVHAAHAEINEAGREVVGS

AEAGVDAASVSEEFRADHPFLFCIKHIATNAVLFFGRCVSP

The proposed best performing SVM algorithms estimates antihypertensive activity of the peptide sequences that have been obtained post digestion process eventuated via combination of human proteases (Chymotrypsin C, Pancreatic Endopeptidase E, Trypsin, Pepsin, Gastricin), which mimics the human gastrointestinal tract digestion.

During the digestion process, the egg white protein breaks into 339 peptides, out of which 37 antihypertensive peptides have been found to be directly matching with the experimentally validated training dataset; while the remaining peptides have been seeded into pre-trained SVM models for predicting the antihypertensive activity (refer to Supplementary File S3). From the set of peptides mentioned in Supplementary File S3.2, the reference SVM model has found 28 antihypertensive peptides, while SIDR (p=0.05) and MRMR ∪ SIDR have found 24 and 27 antihypertensive peptides, respectively, (refer to Supplementary Files S3.3–S3.5). The aforementioned results indicate that the SVM models have bagged the reasonable number of peptides into the antihypertensive category, which is also inline with the experimental findings which suggests that the chicken egg white holds a potential blood pressure lowering effect and its consistent consumption has vital implications for the patients.

## 6. Conclusions

The impact of COVID-19 has been largely observed in the patients suffering from hypertension. The frequent use of drugs that have an ACE inhibitory property can result into upregulation of ACE-II, which contributes to facilitating COVID-19 infection. In this paper, we have proposed to abate the frequency of drug intake and adopt a diet constituting optimal macronutrients. To estimate the food-derived antihypertensive peptide, a nontrivial feature selection and machine learning approach have been suggested which can predict natural hypertension controllers and minimise the intake of ACE inhibitory drugs. The ACE inhibitory peptide database containing 715 peptides is used in this study which have been experimentally validated through in vivo and in vitro models. The maximum attainable accuracy and specificity of the SIDR (p=0.05) SVM models in predicting the antihypertensive food-derived peptide is 86.17% and 84.31%, respectively. The priority of the study has been set on detecting positive food derivative peptides which are directly correlated with statistical Type I error, and the well known metrics which give more emphasis on minimising the error are specificity and AUC. The proposed MRMR ∪ SIDR algorithm is able to achieve the specificity of 85.78% and AUC of 0.9905 without degrading the overall accuracy of the model. The proposed algorithms are expected to help clinicians or patients for personalised decision making for the diet food preferences to constrain the adverse consequences of hypertension.

## Figures and Tables

**Table 1:** Comparison of accuracy of machine learning models for antihypertensive peptides database using Bayesian optimisation routine.

Machine Learning Algorithms	Variants	Accuracy (%)	AUC
Decision trees	Fine	76.9	0.66
	Coarse	80.6	0.65
Logistic regression	-	80.1	0.66
Support vector machine	Linear kernel	80.1	0.63
	Quadratic kernel	80.4	0.66
	Cubic kernel	77.8	0.64
	RBF kernel	81.0	0.68
*k*-nearest neighbour	Fine	78.2	0.63
	Cosine	80.7	0.66

**Table 2:** The *p*-values of all the features demonstrating the statistically significant difference between hypertensive and anti-hypertensive peptide.

Features		*p*-Value	Significant
PseAAC	A (alanine)	0.6881	No
	C (cysteine)	0.0023	Yes ^†^
	D (aspartic acid)	0.8265	No
	E (glutamic acid)	9.2421 × 10${}^{-4}$	Yes ${}^{†}$
	F (phenylalanine)	0.4242	No
	G (glycine)	4.3718 × 10${}^{-14}$	Yes ${}^{†}$
	H (histidine)	0.4542	No
	I (isoleucine)	0.8942	No
	K (lysine)	0.1785	No
	L (leucine)	0.8502	No
	M (methionine)	0.9626	No
	N (asparagine)	0.3234	No
	P (proline)	0.0873	No
	Q (glutamine)	0.6676	No
	R (arginine)	0.1939	No
	S (serine)	0.3363	No
	T (threonine)	0.8461	No
	V (valine)	0.5726	No
	W (tryptophan)	0.0066	Yes ${}^{†}$
	Y (tyrosine)	1.0596 × 10${}^{-4}$	Yes ${}^{†}$
	Sequence order effect	0.0142	Yes *
Structural	Molecular weight	0.0210	Yes *
	R	0.0301	Yes *
	α	0.0723	No
	β	0.8902	No
	$q_{+}$	0.0016	Yes ${}^{†}$
	$q_{-}$	0.3122	No
	Volume	0.0138	Yes *

^†^ For *p* = 0.01 and *p* = 0.05, * For *p* = 0.05 only.

**Table 3:** Estimation of highest accuracy using Bayesian optimisation routine.

Features	Best Accuracy (%)
Reference value (Entire space)	81.0
PseAAC	82.6
Structural	84.5
MRMR	82.2
SIDR (p=0.01)	83.5
SIDR (p=0.05)	85.0
MRMR ∩ SIDR	83.2
MRMR ∪ SIDR	84.9

**Table 4:** Comparison of highest attainable performance of SVM models using a systematic combination search algorithm.

Performance Metrics	Reference Value	PseAAC	Structural	MRMR	SIDR		MRMR ∩ SIDR	MRMR ∪ SIDR
					*p* = 0.01	*p* = 0.05		
Accuracy (%)	84.91	85.47	85.33	84.49	84.07	**86.17**	84.07	**85.61**
AUC	**0.9966**	0.9769	0.9531	0.9093	0.7118	0.8718	0.7621	**0.9905**
Sensitivity (%)	63.15	55.17	87.50	68.18	**86.66**	**85.29**	73.91	80.76
Specificity (%)	84.02	84.19	83.38	82.56	82.45	**84.31**	82.82	**85.78**
MCC	0.2880	0.2738	0.3252	0.2233	0.2524	**0.3774**	0.2551	**0.3728**

In each row, the top two performing metrics have been represented in boldface.

## Data Availability

Not applicable.

## Supplementary Materials

The following supporting information can be downloaded at: https://www.mdpi.com/article/10.3390/nu14142794/s1, File S1: Biological significance of non-trivial features; File S2: Area under curve (AUC); File S3: Peptide fragments of egg white protein. References [56,57,58,59] are cited in the supplementary materials.
