# Supplementary material for: Hypertension: Constraining the Expression of ACE-II by Adopting Optimal Macronutrients Diet Predicted via Support Vector Machine
Source: Nutrients. 2022 Jul 7;14(14):2794. doi: 10.3390/nu14142794 (PMC9318145; doi:10.3390/nu14142794)
Supplement: Supplementary file 1 [file nutrients-14-02794-s001.zip › nutrients-1769025-supplementary.pdf]

Hypertension: Constraining the expression of ACE-II by adopting optimal macronutrients diet predicted via support vector machine
Supplementary file

Mohammad Farhan Khan*, Gazal Kalyan†, Sohom Chakrabarty‡, M. Mursaleen◇

*Digby Stuart College, University of Roehampton, London - SW15 5PU, United Kingdom
†Department of Pathology, School of Medicine and Health Sciences, University of North Dakota, Grand forks - 58202, United States
‡Department of Electrical Engineering, Indian Institute of Technology Roorkee, Roorkee - 247667, India
◇Department of Medical Research, China Medical University Hospital, China Medical University (Taiwan), Taichung - 40402, Taiwan

Corresponding author, M. Mursaleen: mursaleenm@gmail.com

S1: Biological significance of non-trivial features

The statistical analysis has indicated the role of some vital amino acids and structural features in significantly differentiating the properties of the peptides. This section is dedicated to discuss the possible biological significance of some of the non-trivial amino acids and structural characteristics that are significantly contributing to the MRMR and SIDR algorithms in predicting the antihypertensive activities of the peptides [1, 2].

Cysteine: Cysteine is a very unique amino acid which takes part in formation of di-sulphide bond (the only covalent bond formed depending on the environment), and the presence of this amino acid plays significant role in enhancing the antihypertensive activity of the peptides.

Glutamic acid: The presence of Glutamic acid plays significant role in increasing the affectivity of the antihypertensive peptides. It is very interesting to observe that, while presence of glutamic acid plays very critical role whereas aspartic acid with similar charge and nearly similar size doesn't contribute much. It could be thought that presence/absence of a extra carbon plays key factor here. It is also being observed in case of protein biochemistry studies where serine to glutamate mutation could potentially work as phosphomimic whereas aspartate could not perform the same role [3, 4].

Glycine: The unique Glycine is the only achiral amino acid which contains only one hydrogen as its side chain. Presence of the only hydrogen allows huge amount of conformational flexibility in glycine which is known from the comparative ramachandran plot of poly glycine with other amino acids. Due to this unique situation, glycine can reside in parts of protein structures that are forbidden to all other amino acids. The uniqueness of Glycine also means that it can play a distinct functional role, such as using its sidechain-less backbone to bind to phosphates; which means that if one sees a conserved glycine changing to any other amino acid, the change could have an impact. Especially in a small peptide setup presence of a glycine could contribute hugely to the overall flexibility and structural architecture of the candidate molecule. This reflects on the $p-$value in the current study.

Proline The unique cyclic structure of proline's side chain provides itself an exceptional conformational rigidity compared to all other nineteen essential amino acids. This locked structure also affects the rate of peptide bond formation between proline and other amino acids. When proline is bound as an amide in a peptide bond, its nitrogen is not bound to any hydrogen, meaning it cannot act as a hydrogen bond donor, but can only be a hydrogen bond acceptor. Due to the above mentioned conformational rigidity of proline is often found at the end of helix or in turns or loops. Unlike other amino acids which exist almost exclusively in the trans- form in polypeptides, proline can exist in the cis-configuration in peptides. The cis and trans forms contain nearly similar energy.

Tryptophan: Tryptophan contain a six membered ring fused with a five membered ring in its side chain. Because of its bulkier structure of the side chain Trp plays role like a buttress, it's mutation creates local denaturation of the protein structure. In peptide its presence or absence makes huge difference especially on the dynamic property of the peptide which is reflected on the calculated $p-$value.

Tyrosine Tyrosine plays important role because of two reasons. One, it is having an aromatic moiety as a side chain, that allows the amino acid to accommodate in a nearly two-dimensional (2D) space, also it could maintain equilibrium between aromatic and quinone resonating structures.

Sequence order effect: Sequence order effect definitely contributes significantly to the structure and dynamism of the peptide. For example two arginine side by side create steric repulsion not allowing many of the di-hedral rotamers whereas Arg with a Gly behaves very differently.

Molecular weight: While comparing the molecular weight and IC50 values with of the anti-hypertensive peptide in the data-set, it has been found that higher molecular weight peptides contributed significantly to the negative data-set while the positive data-set peptides had lower to medium molecular weight.

R: The shape of the peptide has been actually represented by (R, α, β), where R defines the euclidean distance between the first and last atom. This parameter portrays the fit of the peptide in the active site pocket, thus rendering meaningful for inhibition of the enzyme.

q_+**:** Positive charges of the peptide like to contribute significantly to the property of the peptide, whereas in case of positively charged amino acids like lysine which is not having any significant contribution at all.

Volume The volume of a peptide is the 3D representation of atoms constituting the molecule. The Van der Waal's force between atoms allows them to occupy the space and define the excluded volume of a molecule. The active site of the enzyme ACE-1 has been studied and it was seen that the pocket where the peptide inhibitors bind has two linked sub-pockets with volumes 1181.8Å and 484.8Å. Therefore, the availability of space in which the inhibitor may place must support these limits.

α**:** The shape representation of the peptide also defined by one of the most important parameter i.e. α which defines angle of the atoms. Like R this parameter also correlates the fitting of the peptide to the active site pocket hence contributing significantly to the peptide property.

References

[1] G. C. Barrett, and D. T. Elmore, "Amino acids and peptides", Cambridge University Press: Cambrigde, 1998.

[2] G. Rosenthal, "Plant nonprotein amino and imino acids: Biological, biochemical, and toxicological properties", Academic Press: New York, 1982.

[3] S. Hazra, A. Szewczak, S. Ort, M. Konrad, and A. Lavie, "Post-translational phosphorylation of serine 74 of human deoxycytidine kinase favors the enzyme adopting the open conformation making it competent for nucleoside binding and release", Biochemistry, vol. 50, no. 14, 2011, pp. 2870-2880. DOI: 10.1021/bi2001032

[4] T. McSorley, S. Ort, S. Hazra, A. Lavie, and M. Konrad, "Mimicking phosphorylation of Ser-74 on human deoxycytidine kinase selectively increases catalytic activity for dC and dC analogues", FEBS Letters, vol. 582, no. 5, 2008, pp. 720724. DOI: 10.1016/j.febslet.2008.01.048

S2: Area under curve (AUC)

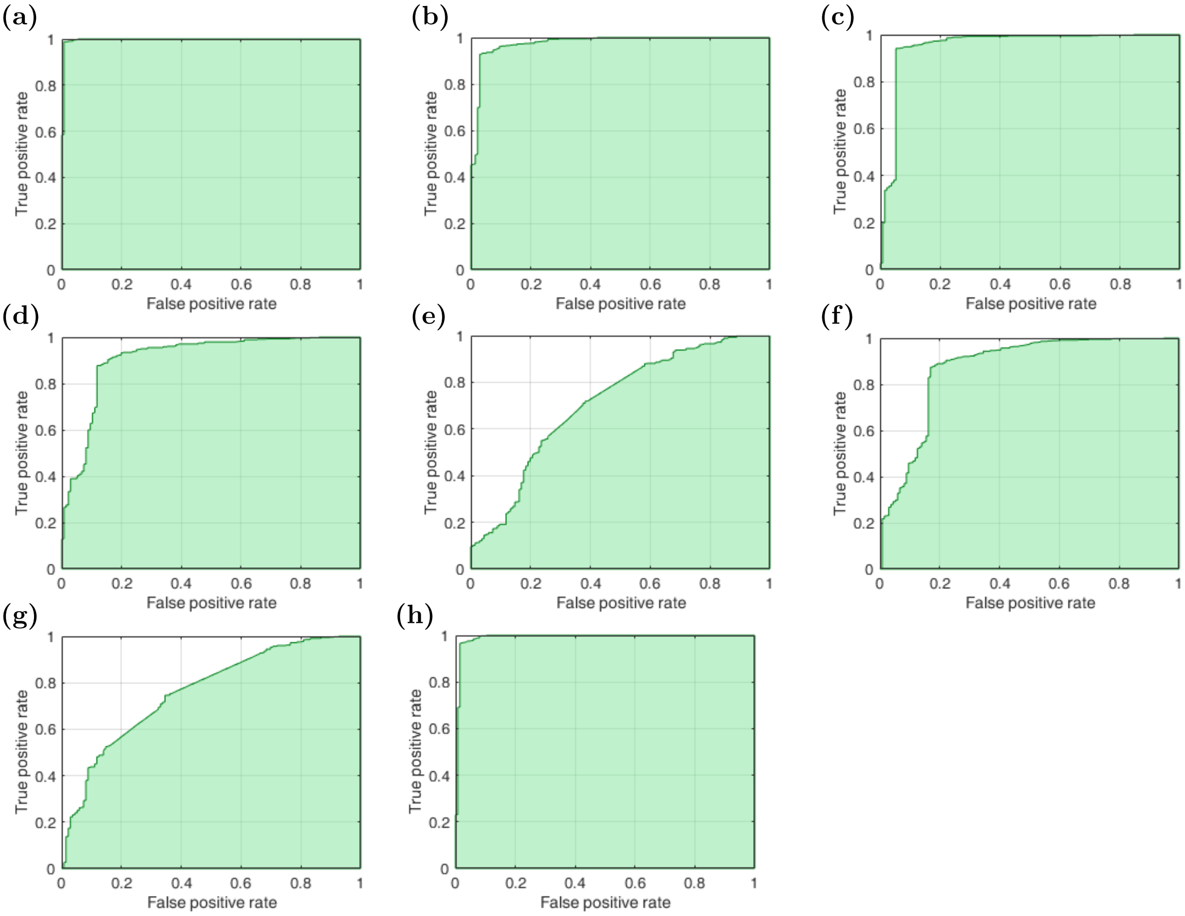

Figure S1: AUC for best performing combination of box constraint ($\hat{C}$) and kernel scale (σ) for the following features: (a) All features (or reference feature space), (b) PseAAC features, (c) Structural features, (d) features extracted from MRMR, (e) features extracted from SIDR ($p = 0.01$), (f) features extracted from SIDR ($p = 0.05$), (g) features extracted from MRMR $\cap$ SIDR, and (h) features extracted from MRMR $\cup$ SIDR

S3: Peptide fragments of egg white protein

S3.1: Peptides matching with experimentally validated training set

RA, GR, GA, AR, YL, IVF, LY, YPIL, HA, PR, RADHP, EK, EA, MA, YAEER, AVL, AV, YR, FR, FY, ADHP, IF, CF, KG, KE, SW, SF, ADHPF, YA, TQ, TE, VL, VM, VY, IG, FL, RADHPF

S3.2: Peptides seeded into SVM models for prediction

SEKMKIL, SFSLA, SVSEEF, KVHHAN, PDEVSG, EAGR, SSSAN, EKAF, VNA, FRVTEQESKPVQMM, ADHPFL, LFFGRCVSP, RGGLEPINFQTA, TAM, KIKV, LKISQA, DQA, SGTMSML, MKMEEKYNL, LELP, MGSIGAASME, VASMASEKMKIL, LFRVASMASEKMKI, LSGISSAES, KVHHA, FKDEDTQAMP, ITKPN, IMSA, KIL, WVESQTNGIIR, NLTSV, QIGLFRVA, CVSP, KISQAVHAAHAEINEAGR, MAMGIT-DVFSSSANL, LESIIN, EN, TSV, PGFGDSIEA-QCGTSVNVHSSL, LFRVA, PILPEY, PDEVSGL, LKISQAVHAAHAEINEAGREVVGSAEAGVDAASVSEE, EPINFQTA, FSSSA, PF, EINEA, DHPFL, FCFDV, CPIA, MV, GSIGAASM, PEYL, IGL, SRLY, GVDA, TAADQ, RELINSWVESQTNGIIRN-VLQPSSVDSQTA, INS, YLGA, MP, KDSTR, NVL, KVHHANENIFY, FQTAADQARE, EKL, GIIRN, PEY, VESQ, MVLVNA, DVF, EKA, TSSN, ESIIN, DVY, REL, TSVL, EVVGSAEAGVDAASVSEE, SME, FLFCIKHIA, SMA, MGSIGA, SGISSAESL, WTSSNVMEER, GGL, QCVKEL, MVY, VTE-QESKPVQMMYQIGL, SRL, MKMEEK, SMEFCFDVFKEL, LTE, AVHAAHAEIN, ESIINFEKL, MVL, KVHHANENI, VNAIV, GDSIEAQ, SR, VASMASEKMKI, CIKHIATN, SM, SL, LMA, SA, DIL, AIV, NENIFY, CGTSVN, LKVHHANENI, FDKL, NLTSVLMA, KIKVYL, TEWTSSNVMEER, CVKEL, EIN, PGF, SVSEEFRA, ASGTM, LA, ASRL, GAKDSTRTQ, EVVGSAEAGVDAASVSEEFR, ESLK-ISQA, GDSIEA, RGGL, DQ, VTEQ, VA, VN, IV, KDSTRTQ, AEER, IM, IN, MKMEEKY, WEKA, WTSSNVMEERKIKV, KDSTRTQINKVVRFDKLPGFGDSIEA, LAMV, ARE, KISQ, EQL, GIIR, CVKE, LINS, LKVHHA, CIKHIA, MVYL, GAKDSTR, EERKIKVY, SGTMSM, DKL, SVSEE, SEKMKI, FSSSAN, RVA, PIL, SEKMKILELPFA, SAL, PRM, QIG, VTEQESKPVQMM, RE, FCIKHIA, VTE-QESKPVQMMY, KDEDTQ, QPSSVDSQTA, SSSA, QCVKE, QCGTSVNVHSSLRDILNQITKPNDV, KVVR, FQTA, EERYPILPEY, FLFCIKHIATNAV, QCGTSVNVHSSLRDILNQITKPNDVY, CPIAIM, LASRL, TQINKVVR, FFGR, SFS, FCIKHIATNAV, SFSL, LPRMKMEEKY, ESKPVQ, MGITDV, EER, QPSSVDSQTAMVL, EEK, AEERY, RVASM, PFASGTMSML, GRCVSP, TNA, LSGISSA, EEKY, KISQA, CPIAIMSAL, IVFKGL, IVFKGLWEKA, RGGLEPIN, AMV, EL, SGTM, PGFGDSIEA, AM, EF, WEKAFKDEDTQAMPFR, FKE, DHPF, LWEKA, AIVF, FKG, EQ, ES, GITDV, NQITKP-NDVYSFSL, SGTMSMLVLLPDEVSGLEQLESIINFEKLTEWTSSNVMEERKIKVY, LQCVKE, YL-PRMKMEEK, LMAMGITDV, MAMGITDV, LQCVKELY, KIKVY, NQITKPNDVY, QIGL, ASEKM, EERY, VHSSL, WVESQTNGIIRNVLQPSSVDSQTAMV, CPIAIMSA, EAGREVVGSAEAGVDAASVSEEF, MGITDVFSSSA, EERKIKV, YNLTSV, ASR, NL, AREL, DHPFLFCIKHIA, SEKM, FYCPIAIMSA, NLSGISSA, TEW, NQITKPNDV, FYCPIA, FEK, GITDVF, EAGREVVGSAEAGVDAASVSEE, QPSSVD-SQTAMV, WVESQTNGIIRNVLQPSSVDSQTA, SGISSA, KEL, SMEFCFDVFKELKVHHA, AMVY, KI, LPRMKMEEK, KM, LEQ, FA, EPIN, MPFR, YSFS, GREVVGSA, YNL, FRADHP, EPINFQ-TAADQAR, YQIG, FRA, RDIL, FCIKHIATNAVL, DV, INSWVESQTNGIIR, VHA, FKDEDTQA, RVTEQ, LGAKDSTRTQINKVVRFDKLPGFGDSIEAQCGTSVNVHSSLRDILNQITKPNDV, LVNAIV, PSSVDSQ, TN, LVLLPDEVSG, EVVGSAEAGVDAASVSEEF, MPFRVTEQESKPVQMMY, QCGTSVN-VHSSL, LYRGGLEPIN, TA, GSIGA, VASM, NENIFYCPIA, FASGTMSM, LVNA, VNAIVFKGL, NENI, ESL, DHP, PFA, KVVRF, LGA, EVVGSA, FQTAADQAR, KGL, SGISSAES, SVSEEFR

S3.3: Antihypertensive peptides predicted using reference SVM

ADHPFL, AEER, AEERY, CPIAIM, CPIAIMSA, CPIAIMSAL, CVKE, CVKEL, EQ, EQL, FYCPIA, FYCPIAIMSA, INS, KVVR, KVVRF, LA, LAMV, LASRL, LELP, LTE, LVLLPDEVSG, MP, MPFR, PF, PFA, PFASGTMSML, SVSEEFRA, SVSEEFRDVY

S3.4: Antihypertensive peptides predicted using SIDR($p = 0.05$) algorithm

ADHPFL, AEER, AEERY, CPIAIM, CPIAIMSA, CPIAIMSAL, CVKEL, EQ, EQL, FYCPIA, FYCPIAIMSA, KVVR, KVVRF, LAMV, LASRL, LELP, LTE, MP, MPFR, PF, PFA, PFASGTMSML, SVSEEFRA, SVSEEFRDVY

S3.5: Antihypertensive peptides predicted using MRMR $\cup$ SIDR algorithm

ADHPFL, AEER, AEERY, CPIAIM, CPIAIMSA, CPIAIMSAL, CVKE, CVKEL, EQ, EQL, FYCPIA, FYCPIAIMSA, INS, KVVR, KVVRF, LA, LAMV, LASRL, LELP, LTE, LVLLPDEVSG, MPFR, PF, PFA, PFASGTMSML, SVSEEFRA, SVSEEFRDVY
